# DiscMycoVir: a user-friendly platform for discovering mycoviruses in fungal transcriptomes

**DOI:** 10.1186/s12859-025-06196-z

**Published:** 2025-07-07

**Authors:** Agorakis Bompotas, Nikitas-Rigas Kalogeropoulos, Maria Giachali, Ioly Kotta-Loizou, Christos Makris

**Affiliations:** 1 Department of Computer Engineering and Informatics, University of Patras, 26504 Rion, Achaia Greece; 2 Department of Clinical, Pharmaceutical and Biological Science, School of Health, Medicine and Life Sciences, University of Hertfordshire, Hatfield, AL10 9AB UK; 3 Department of Life Sciences, Faculty of Natural Sciences, Imperial College London, London, SW7 2AZ UK

**Keywords:** Mycovirus discovery, Sequence analysis, Pipeline, Docker

## Abstract

**Purpose:**

The article presents DiscMycoVir, an elegant and user-friendly platform for discovering mycoviruses in fungal transcriptomes. DiscMycoVir is a pipeline of established tools for next-generation sequencing analysis and database searching, incorporated in an interface that facilitates accessibility even for users that have no programming skills and expertise. A comprehensive and detailed result report enhances user experience. DiscMycoVir can be accessed online for reviewing purposes at: https://discmycovir.imslab.gr:8000 and the source code is located at https://github.com/abompotas/DiscMycoVir. We recommend using the GitHub repository, as the online platform may lack the necessary resources to ensure uninterrupted service especially on large files.

**Methods–results:**

We employed state-of-the-art technologies in the design and implementation phase of the platform. We present the application of the platform in screening RNA-seq data from the yeast *Candida auris* for mycoviruses, demonstrating its efficiency and simplicity in use.

**Conclusions:**

DiscMycoVir serves as a user-friendly platform for identifying mycoviruses in RNA-seq data. Our tool was successfully implemented to discover mycoviruses in a *C. auris* isolate and could be adapted to detect viruses in transcriptomes from other organisms as well.

## Background

Viruses are obligatory parasites infecting all living organisms, including fungi. Fungal viruses or mycoviruses have been detected in most major taxa of fungi, including ascomycetes and basidiomycetes together with early diverging lineages [1]. Mycovirus infection often leads to phenotypic alterations in the fungal host, including but not limited changes in morphology, pigment production, sporulation, growth, virulence, pathogenicity and drug resistance [2]. In light of this, it is evident that mycovirus infection is ecologically, medically and economically significant with impact in agricultural, medical and biotechnological settings. Therefore, mycovirus diagnostics to determine presence of already known and/or novel mycoviruses in fungal isolates or populations is crucial.

Traditionally, mycoviruses are detected through the presence of double-stranded (ds) RNA in the fungal host. The vast majority of mycoviruses have RNA genomes, therefore such dsRNA elements correspond to either the genome of a dsRNA mycovirus or the replication intermediate of a single-stranded (ss) RNA virus and are considered hallmarks of mycoviral infection. However, the development of next-generation sequencing (NGS) technologies, such as RNA-seq, facilitated and accelerated discovery of novel mycoviruses, particularly those with positive-sense (+) or negative-sense (–) ssRNA genomes that may be present in low quantities below the detection limit of traditional methods. Using bioinformatics to detect mycoviruses in fungal transcriptomes generated by RNA-seq is not uncommon amongst mycovirologists but requires substantive computational knowledge and skills.

Access to comprehensive databases and repositories is crucial for bioinformatics research. The National Centre for Biotechnology Information (NCBI) hosts a variety of databases relevant to biotechnology and biomedicine, providing a wealth of information for researchers. Of particular interest is the NCBI Sequence Read Archive (SRA), the largest publicly available repository of high-throughput sequencing data from fungi among other organisms together with metagenomic and environmental surveys. Evidence of mycovirus infection may be detected in SRA files by examining reads that do not originate from the genome of the fungus under study. Annotated genome sequences for a range of fungi are publicly available in NCBI Genome, while Ensembl [3] and in particular Ensembl Fungi is another valuable resource, offering a genome browser with access to annotated genomes.

One of the primary areas in bioinformatics is sequence analysis. The NCBI Basic Local Alignment Search Tool (BLAST) [4] is widely used for comparing nucleotide or protein sequences to sequence databases and identifying those that are significantly similar. BLAST+, a suite of command-line tools to run BLAST, may be used to detect sequences homologous to known (myco)viruses among those that do not have a fungal origin. However, the process of reliably obtaining this information from raw data is complex and requires either knowledge of computer programming or user-friendly platforms for researchers without programming skills, such as Galaxy and SEquence DAtaset builder (SEDA).

Galaxy [5] is the main web-based platform for omics data analysis, including genomics and transcriptomics, but also proteomics and metabolomics. Galaxy provides a user-friendly interface and supports the integration of various tools and resources, offering versatility for numerous applications. Galaxy ensures reproducibility by tracking all steps and parameters used, rendering it easy to replicate data analyses. Accessible from anywhere with internet access, Galaxy is open-source and supported by a strong community and extensive documentation.

SEquence DAtaset builder (SEDA) [6, 7] is a cross-platform desktop application designed to manipulate FASTA files containing nucleic acid or protein sequences. SEDA features a user-friendly graphical interface that includes basic utilities like filtering, sorting, and reformatting files, together with advanced tools such as BLAST, gene annotation, protein domain annotation, and sequence alignment-capabilities often missing in similar software. SEDA facilitates efficient management of large datasets and offers easy-to-install distributable versions, including a Docker image for Linux.

Despite their numerous strengths, Galaxy and SEDA exhibit significant complexity in their installation and maintenance, requiring a level of technical expertise that may be daunting for inexperienced users or those without a strong background in bioinformatics or computer science. Despite their numerous strengths, Galaxy and SEDA exhibit significant complexity in their installation and maintenance, requiring a level of technical expertise that may be daunting for inexperienced users or those without a strong background in bioinformatics or computer science. The extensive range of tools and options available may be overwhelming for inexperienced users, while understanding the appropriate tools and workflows requires a steep learning curve. Another challenge lies in their scalability, as handling large datasets or intensive computations may strain server resources, leading to performance bottlenecks or slow processing times. Moreover, their dependencies on third-party tools and plugins may introduce compatibility issues or vulnerabilities, potentially compromising data integrity or security

*Our contribution* Here we aim to provide a simple user-friendly platform for mining viral sequences from transcriptomics data. We term our platform ‘Mycovirus Discovery’ or ‘DiscMycoVir’ since its initial use, described in the present article, is for discovering mycoviruses in fungal transcriptomes. DiscMycoVir is purpose-built, ready-to-use and accessible to users with no programming skills or expertise in RNA-seq data analysis, emphasising on a comprehensive and detailed result-reporting experience. To the best of our knowledge, this is the first and only publicly available platform for mycovirus discovery.

## Materials and methods

In this section, we present the resources and methodologies employed for developing and applying the DiscMycoVir pipeline. We describe both the biological data processing techniques and the technical components that enable automated mycovirus detection.

### Bioinformatics software tools

FASTQC 0.12 [8] is used for quality control of the raw reads. FASTQC provides a comprehensive report in HTML format, which integrates seamlessly with various web development frameworks, allowing for easy implementation within our pipeline. By offering both summary statistics and detailed visualisations, FASTQC assists the user in identifying potential issues in the data before proceeding with downstream analyses. Such issues are addressed by Trimmomatic 0.36 [9] that processes raw reads as instructed by the user, enhancing overall quality, and whose command line interface can be easily added in our pipeline. The default processing includes clipping adapter sequences, trimming low quality bases using a sliding window, and filtering short processed reads. However, Trimmomatic is highly configurable, allowing the user to customise parameters according to their specific needs.

Following quality control, Trinity RNA-seq 2.11 [10] is used for the *de novo* assembly of contigs, representing transcripts, from the processed reads without the use of a reference sequence. Subsequently, Burrows-Wheeler Aligner (BWA) [11] and specifically BWA-MEM v0.7.17 [12], which is optimised for high-quality sequences longer than 70 bp and exhibits superior speed, accuracy, and capacity to manage longer reads and higher error rates, is used to align the assembled contigs to a reference genome provided by the user. SAMtools v1.13 is then used for selecting unaligned contigs that do not originate from the fungal genome

Finally, the BLAST+ algorithm [4] is implemented for accessing online databases to compare these unaligned contigs against known sequences to identify regions of similarity. For this study, we utilised the NCBI nt_viruses database [13], which is a comprehensive collection of nucleotide sequences specifically curated for viral research.The default command-line arguments used for Trimmomatic, Trinity, BWA, SAMtools and BLAST+ are summarized in Table 1

**Table 1:**
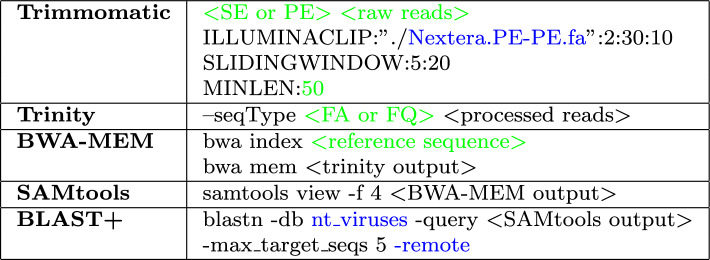
Standard software tool commands

Arguments in green can be modified by user

### Application development technologies

In our development of a bioinformatics application for mycovirus discovery, several robust technologies were employed to ensure efficiency, scalability, and user-friendliness. Key among these technologies are Docker, Flask, Ionic, and Angular, each playing a pivotal role in the system’s architecture and functionality.

Docker [14] is utilised for containerisation, enabling the application to run in isolated environments and ensuring consistency across different stages of development, testing, and deployment. Docker containers encapsulate the application along with all its dependencies, allowing for seamless integration and eliminating compatibility issues. This is particularly crucial in bioinformatics, where the software stack can be complex and diverse. Docker enhances reproducibility, an essential aspect of scientific research, by providing a stable and uniform environment for the application to operate.

Flask [15] acts as the back-end framework, providing a lightweight yet powerful platform for developing the server side of the application. Flask’s simplicity and flexibility make it ideal for handling the intricate data processing and computational tasks required in virus discovery. It supports the integration of various bioinformatics tools and databases, ensuring efficient data management and retrieval. Flask’s capability to handle RESTful APIs facilitates smooth communication between the front-end and back-end components, enabling real-time data exchange and updates.

Ionic [16] serves as the framework for developing the mobile and cross-platform aspects of the bioinformatics application. Its hybrid nature allows the creation of a single codebase that runs on multiple platforms, including iOS, Android, and web browsers. Ionic’s robust set of components and plugins simplifies the development process, enabling a responsive and visually appealing user interface. This is vital for the application’s usability, as it ensures that researchers and clinicians can access and interact with the virus discovery tools seamlessly, regardless of the device they are using.

Angular [17] is employed for building the front-end web application. As a powerful framework maintained by Google, Angular offers a comprehensive solution for developing dynamic and responsive user interfaces. It enables the creation of a sophisticated and intuitive interface that allows the user to interact with complex bioinformatics data effortlessly. Angular’s modular architecture and extensive library support facilitate the integration of advanced features, such as real-time data visualisation and interactive dashboards, which are essential for analysing viral sequences and conducting in-depth research.

Together, these technologies accompanied by a simple MySQL [18] database, form a cohesive and efficient system for mycovirus discovery. Docker ensures a reliable and consistent environment, Ionic and Angular provide a responsive and user-friendly interface, and Flask handles the complex data processing and server-side logic.

## System design

In system design, we outline the complete system and pipeline architecture. We provide a detailed discussion of the application architecture, emphasise the critical elements of our implementation and illustrate how well-known frameworks were utilised to enhance our design.

**Fig. 1:**
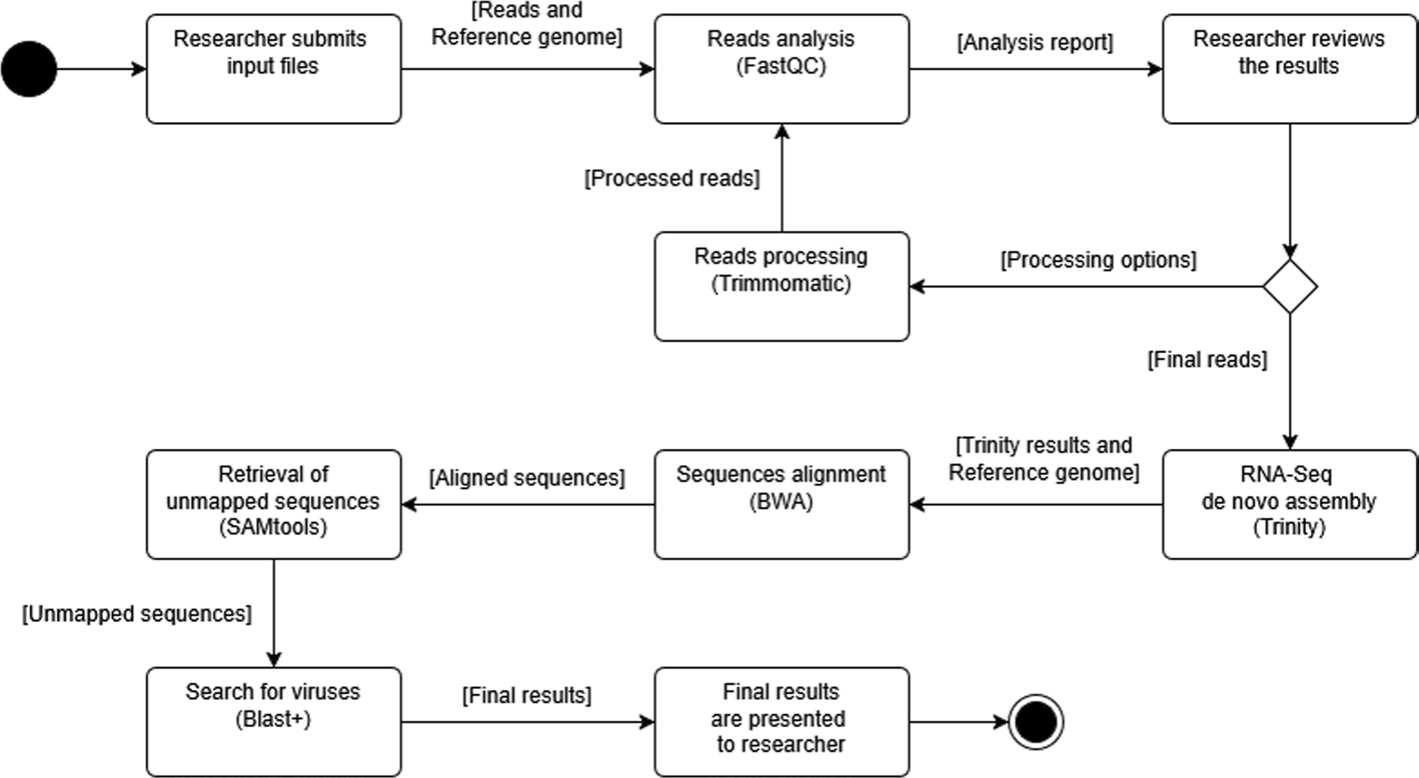
Analysis Process. Once the user creates a job by submitting the files containing the raw reads, a quality control step is performed and the results are communicated to the user via email. The user reviews the results and decides on how to improve the quality of the reads. Once the user is satisfied with the quality of the reads, they submit the job for mycovirus discovery and are notified via email when the job is completed to review the results

The process is initiated when the user submits information, including one or more files containing raw reads from RNA-seq experiments, through the platform’s interface. The reads in the submitted files first undergo quality control and the user is given the option to perform trimming and clipping to remove low-quality and adapter sequences, ensuring that only high-quality data is used for further analysis. Afterwards, the results are reviewed, allowing the user to assess of the data once more.

Following quality control, the processed, the reads are used to produce contigs which are then aligned to the reference genome of the fungus they are derived from, a crucial step for discarding contigs of fungal origin. The non-aligned contigs, which may potentially be originating from mycoviruses, are then subjected to BLAST+ queries to identify and annotate sequences by comparing them against a comprehensive database. Throughout the process, the system automatically notifies the user via email about the progress of the analysis. These emails include links for parameter adjustments or to view intermediate and final results. The whole process is shown in Fig. 1.

**Fig. 2:**
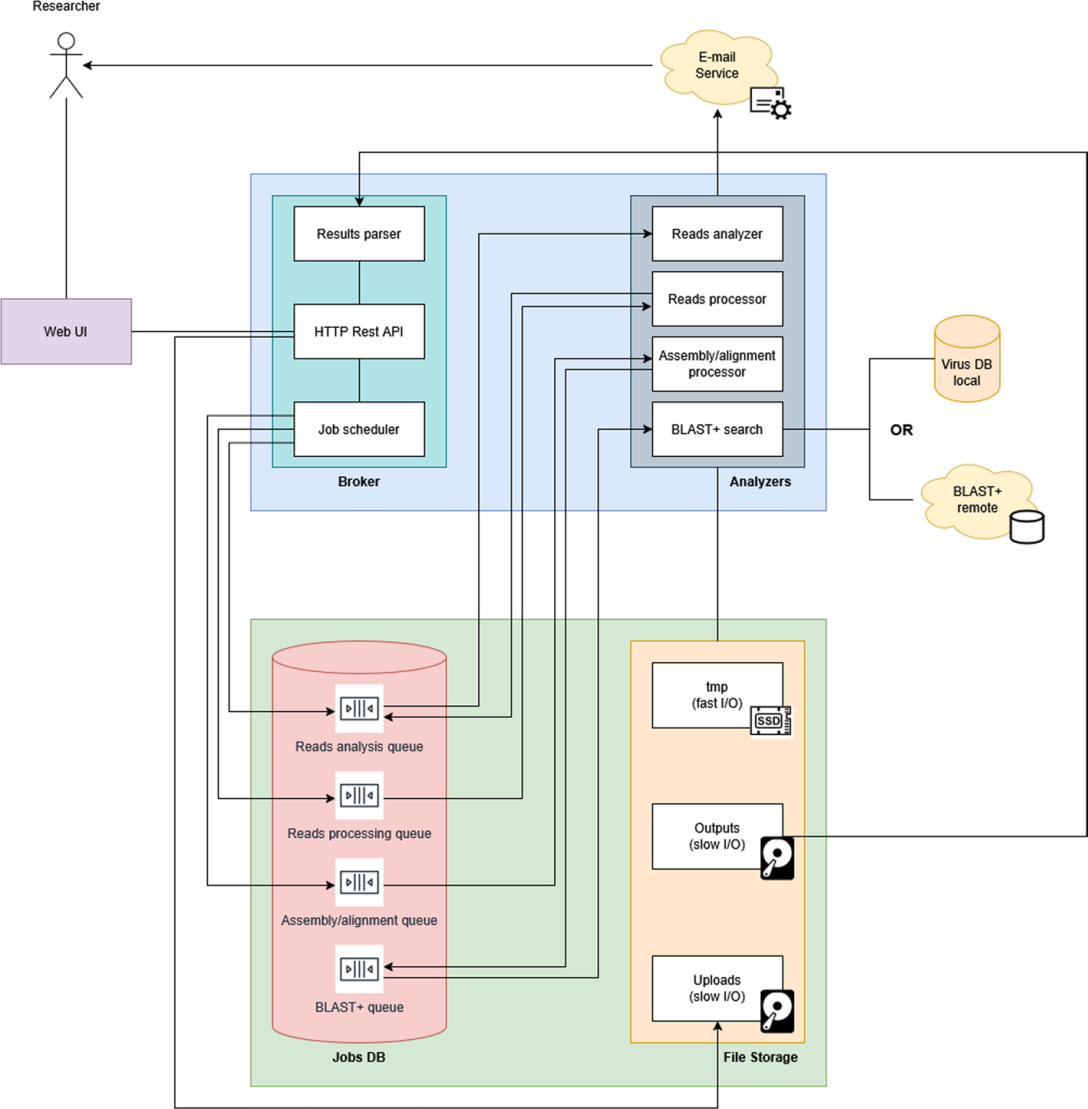
System Overview. The user interacts with the platform via a web user interface and an email service. A broker and an analyser comprise the backend, with the former orchestrating the entire process and the latter being responsible for the data analysis. Information regarding each job and relevant files are stored respectively in JobsDB and files storage

Figure 2 provides an overview of our application. The user, typically a researcher, interacts with the platform through a web user interface, where they can submit the necessary files and information. The system, in turn, updates the user on progress via an automated email process. The content of these emails varies depending on the analysis phase, but they always include a link to the relevant interface, allowing the user to either modify parameters or view a report on the results.

Diving deeper into the application design, each analysis is divided into simpler jobs stored in their respective queues. These queues are implemented with the help of a relational database, which we name JobsDB, and is designed to manage the workflow and metadata associated with virus discovery processes in a bioinformatics environment. It features a primary key (id) for unique experiment identification and timestamps (submitted, started, completed) to track job submission and various stages of analysis (e.g., quality control, read processing, assembly, alignment, BLAST+ query). Essential metadata includes an email address for notifying the user about status changes during the process, sample names and genome details. There are also fields to indicate the format of the input files (FASTA or FASTQ), which may be compressed in.gz format and whether the experiment includes single-end or paired-end sequencing data, while additional information and parameters provided by the user, such as adapter sequences and minimum sequence length, enhance customisation. The files that are uploaded for analysis and those that are created by the system are stored outside the JobsDB in the OS filesystem.

On the backend side of the application, a broker acts as an intermediary between the experiment job analyser and the user via the web interface. This module handles communication with the analyser, the primary component of experimenting. The broker facilitates communication, coordination, and data exchange between the various system components. It can be regarded as a middleware layer that orchestrates the individual tasks, ensures the validity of the user’s inputs and formats the results in a way that is easily parsed by the application’s frontend.

However, the primary and most critical component of the backend is the analyser. It parses the fields of each job in the JobsDB to carry out the appropriate phase of the necessary analysis. After each analysis step, the module notifies the user via email about the status of the analysis and provides a link for parameter adjustments. A significant challenge in such applications is the time required to conduct and complete the analysis. To address this, our approach focuses on dividing the pipeline into independent phases that can be executed asynchronously.

The broker as mentioned before is responsible for orchestrating the entire process, ensuring that tasks are scheduled, executed, and monitored effectively. It consists of three main components the Job Scheduler, the HTTP Rest API and the Result Parser.

- Job Scheduler: This component manages the scheduling of jobs, ensuring that tasks are queued and processed in the correct order.
- HTTP REST API: This API facilitates communication between the Broker and other system components, allowing for data exchange and command execution through HTTP requests.
- Results Parser: After the analysis is completed, the Results Parser processes the output data, extracting relevant information and preparing it for further use or notification.

Considering that each analysis is time-consuming, we integrated separate queues to manage the different stages of the analysis pipeline, instead of one queue for all experiments. The jobs DB is a MySQL-dedicated storage system that contains the necessary information about each job.

- Analysis Queue: Jobs awaiting initial analysis are placed in this queue.
- Processing Queue: After initial analysis, jobs are moved to this queue for processing of raw reads, including trimming, clipping and filtering.
- Alignment Queue: Once processing is complete, jobs proceed to this queue for genome alignment.
- BLAST+ Queue: Finally, jobs are placed in this queue for sequence alignment using the BLAST+ algorithm.

This architecture enables user to make further adjustments to the parameters at various stages of the experiment. The most common adjustment occurs during the processing stage, where user can review the FastQC reports on the raw and processed reads and modify the parameters if necessary.

The File Storage is responsible for managing the various files used and produced during the analysis. It is divided into three sections:

- Uploads: this section stores the original genome files uploaded by researchers. Since these files can be very large and only a few I/O operations are required directly on them they can be stored on a cheap secondary memory.
- Temporary Storage: temporary files generated during the analysis process are stored here. It is recommended to utilise a fast secondary memory system like an SSD for this storage for faster read/write operations. Those files are removed as soon as possible for effective and efficient storage management.
- Outputs: the final output files, containing the results of the analysis, are stored in this section, which the user can download and review locally. Similar to the Uploads storage a slower but cheaper storing method can be used.

## Results and discussion

In this section, we present our web application design and implementation through the lens of a typical biological problem, i.e., a mycovirus discovery case study. We highlight the level of detail in the output presentation and offer guidelines for interpretation.

### Use study: *Candida auris*

*Candida auris* [19] is a yeast and an emerging multidrug-resistant fungal pathogen that has become a significant concern in healthcare settings worldwide due to its high mortality rate and ability to cause outbreaks in hospitals. *C. auris* was first identified in 2009 in Japan from the ear canal of a patient. Infections caused by *C. auris* can range from superficial (such as ear infections) to invasive (such as bloodstream infections), which can be life-threatening, particularly in immunocompromised patients. The mortality rate for invasive *C. auris* infections is high, partly due to the severity of infections in vulnerable patient populations and the pathogen’s resistance to anti-fungal treatments. *C. auris* can form biofilms, which enhance its resistance to anti-fungal treatments, and survive on surfaces and medical equipment for extended periods, contributing to its ability to cause outbreaks in healthcare settings. Given the presence of mycoviruses in other yeasts and human pathogenic fungi, it is plausible that *C. auris* could harbour mycoviruses. Mycoviruses may alter *C. auris* virulence and anti-fungal resistance profiles, for example by affecting host metabolic pathways [2]. We will explore the presence of mycoviruses which might contribute to the genetic diversity of *C. auris* populations, influencing their adaptability and evolution. Understanding the iterations between mycoviruses and *C. auris* could open new avenues for therapeutic interventions.

### Using the DiscMycoVir application

**Fig. 3:**
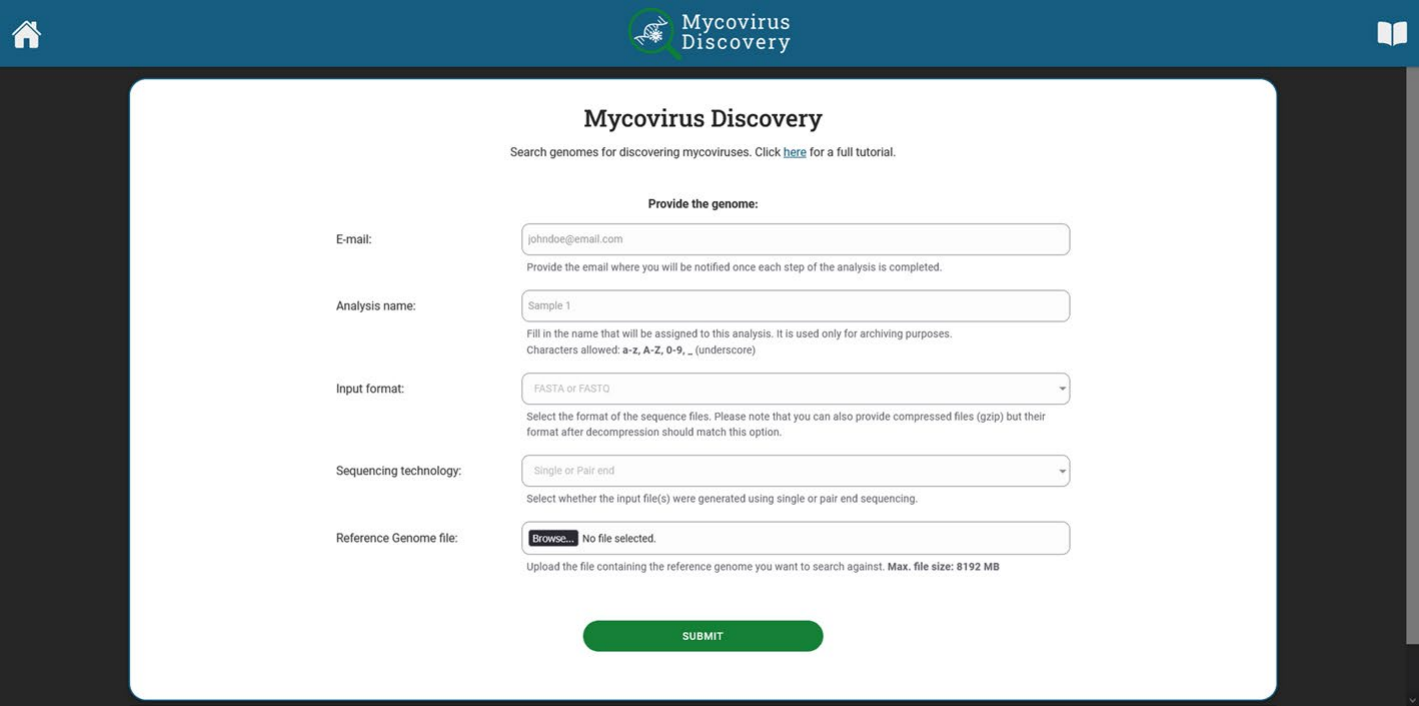
Application Main Screen. The user provides an email address, a name for their analysis, the input format of the raw reads, and the sequencing technology used. The latter determines whether one (single end) or two (paired end) files of raw reads need to be uploaded together with the reference genome file

Figure 3 shows the user interface for the Mycovirus Discovery platform, which features a straightforward form for submitting all required information. The user is prompted to provide their email address, a sample name, the input format of the raw reads (FASTA or FASTQ) the sequencing technology used (singe end, SE or paired end, PE) and a reference genome file. Once these details are filled out, the user clicks the “Submit” button to submit the job to the system for analysis and receives a link to the next phase screen on their e-mail address. For the case study, single end raw reads generated by Illumina HiSeq 250 using a cDNA library constructed from the transcriptome of clinical *C. auris* (SRR11550480), together with the reference genome of *C. auris* strain B11221 (ASM3135756), were uploaded.

**Fig. 4:**
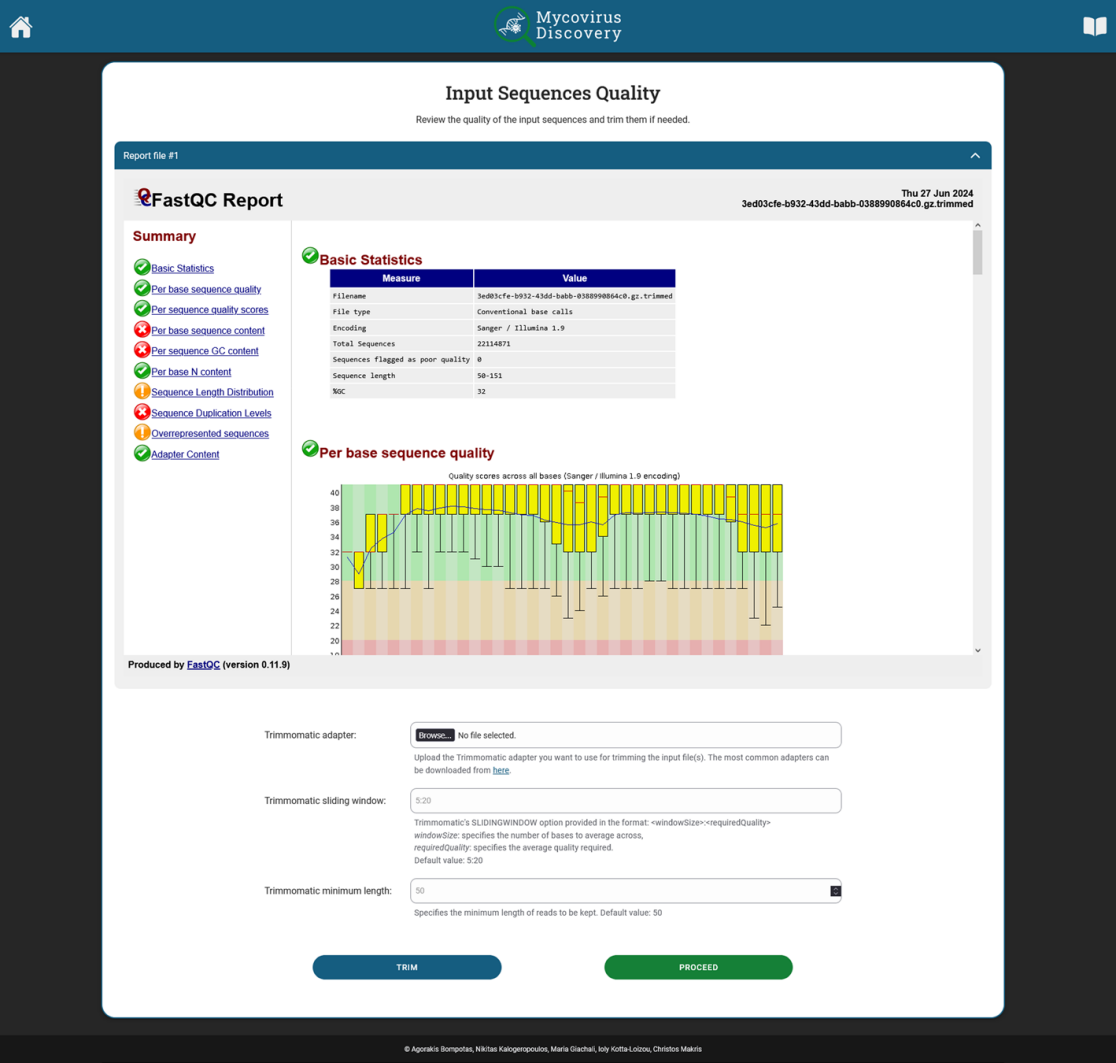
FastQC report. A quality control report on the input file(s) of the raw reads is generated by FastQC and made available to the user. The report includes information on basic statistics, per base sequence quality, per sequence quality score, per base sequence content, per sequence GC content, per base N content, sequence length distribution, sequence duplication levels, overrepresented sequences and adapter content

After the job is submitted and quality control of the reads is conducted, the report screen, shown in Fig. 4, is displayed by following the generated link received in user’s the e-mail. For the case study, only the ‘per base sequence content’ and the ‘sequence duplication levels’ were evaluated as very unusual (red cross) by FastQC, while all other modules were entirely normal (green tick). The former stems from non-completely random binding of the random hexamer primers used for constructing the cDNA library from RNA transcripts and the latter reflects the multiple copies of RNA transcripts originating from certain genes, as expected.

The user then decides whether to process the raw reads to improve their quality or proceed with the next step of the pipeline. If the user is concerned with the quality of the reads, they can modify the processing parameters of Trimmomatic at the bottom of the screen based on the FastQC report. For instance, clipping adapter sequences is facilitated by providing a file with the adapter sequences used for library construction; trimming low-quality bases is performed employing a sliding window approach, where the quality scores within a defined window are assessed and trimming occurs once the average quality score within the window falls below a user-specified threshold; following clipping and trimming, reads below a user-specified length are discarded. This processing ensures that only high-quality portions of the reads are retained, which is crucial for subsequent accurate sequence alignment. By clicking the “Process” button, quality improvement using Trimmomatic is initiated. It is impor- tant to note that the user can implement the quality improvement process on the raw reads until a satisfying report is generated. If the user is satisfied with the quality of the reads, they can proceed to the next stage of the pipeline by clicking the “Proceed” button. For the case study, clipping, trimming and filtering was performed using the default Trimmomatic parameters.

### Presentation and visualisation of findings

**Fig. 5:**
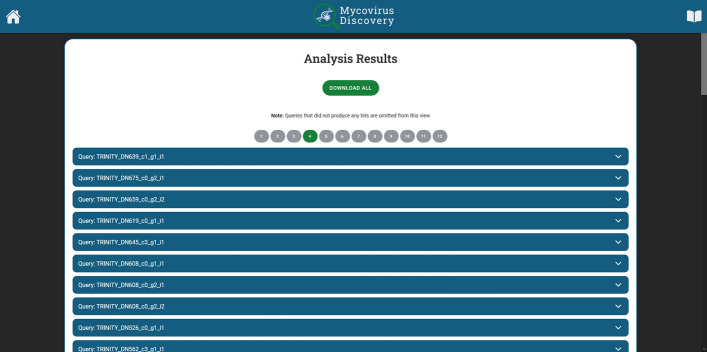
Application Results. A summary list of all contigs that generated an alignment to sequences in the user’s database of choice is available for further investigation by clicking the dropdown panels

**Fig. 6:**
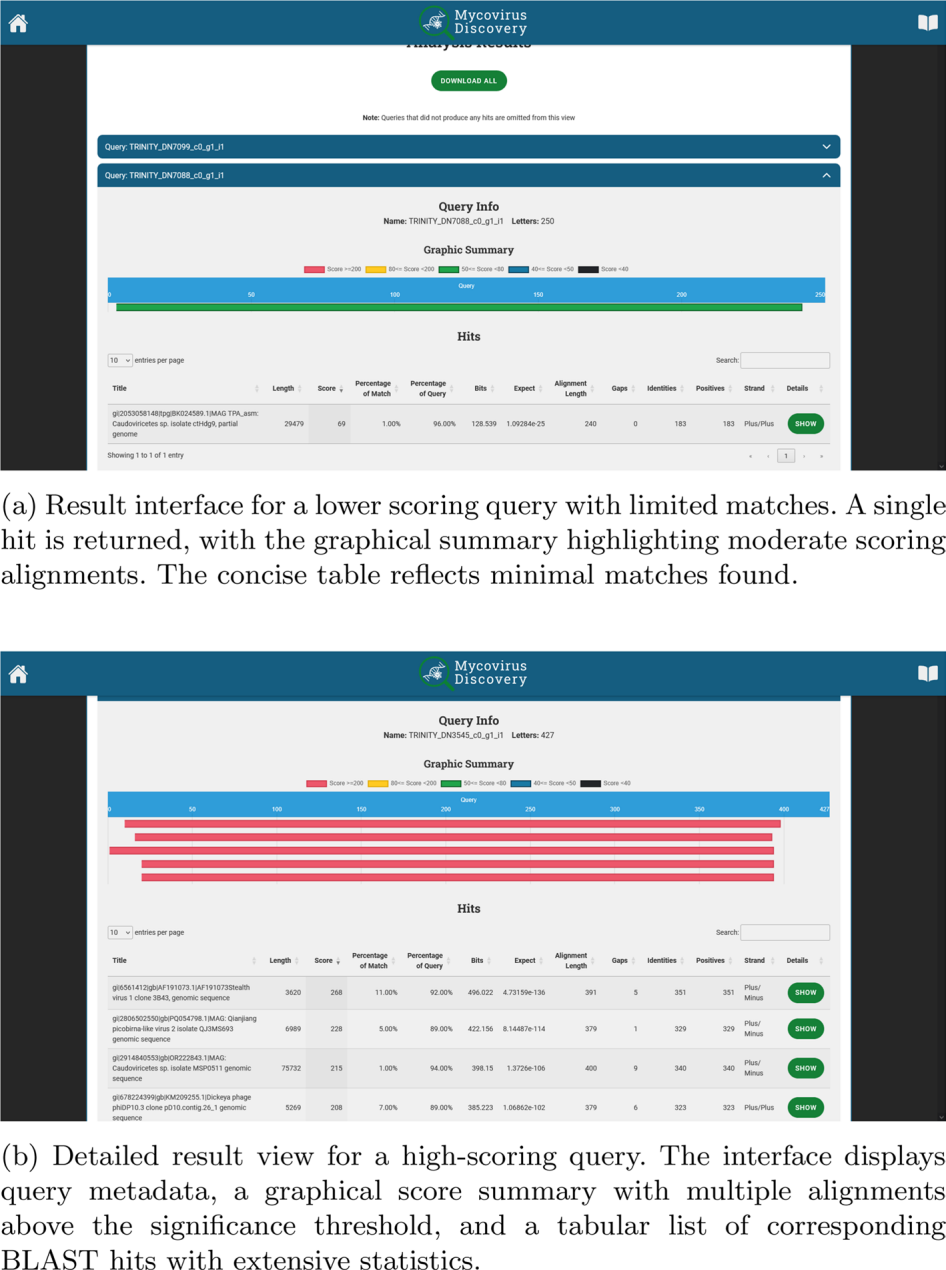
Application Reports. For each contig, the query information, a graphic summary, and a list of hits with all BLAST statistics, together with the percentage of match that shows the coverage of the match sequence by the query sequence, are included. Top panel: result view for a low scoring query with a single match. Bottom panel: result view for a high scoring query with numerous matches

After processed reads are assembled into contigs by Trinity, the contigs are aligned on the fungal genome by BWA and unaligned contigs are selected by SAMtools, an external tool integrated into the system, BLAST+, is used to compare the non-aligned contig sequences against a database of known sequences to identify potential matches. For the case study, the NCBI nt_viruses database was used. Figure 5 shows the generated reports. Each contig match is represented by a dropdown panel, which, when clicked, displays a detailed report (Fig. 6).

In Fig. 6 the specifics of each query match are illustrated. The panel begins with a graphical representation of the query alignment. In the hits section, the user can examine key statistical elements related to the matches. It features a table listing various hits, with columns for title, length, score, bits, expect value, alignment length, gaps, identities, positives, strand, and details. Two examples are illustrated: a low scoring query with a single match (top panel) and a high scoring query with numerous matches (bottom panel). The former is a concise table reflecting minimal matches found for a 250 bp contig that aligns solely to the genome of a Caudiviricetes sp. bacteriophage with moderate scoring as highlighted by the graphical summary. The latter is an extensive table with multiple alignments above the significance threshold for a 427 bp contig that aligns to numerous viral sequences, including cytomegalovirus-related Stealth virus 1, a picobirna-like virus and bacteriophages. These viruses are not promising candidates for infecting *C. auris*, since their known hosts are bacteria or animals, and may be the outcome of sample contamination. Further investigation is required, and users can explore individual matches in detail by clicking on the green “show” button.

**Fig. 7:**
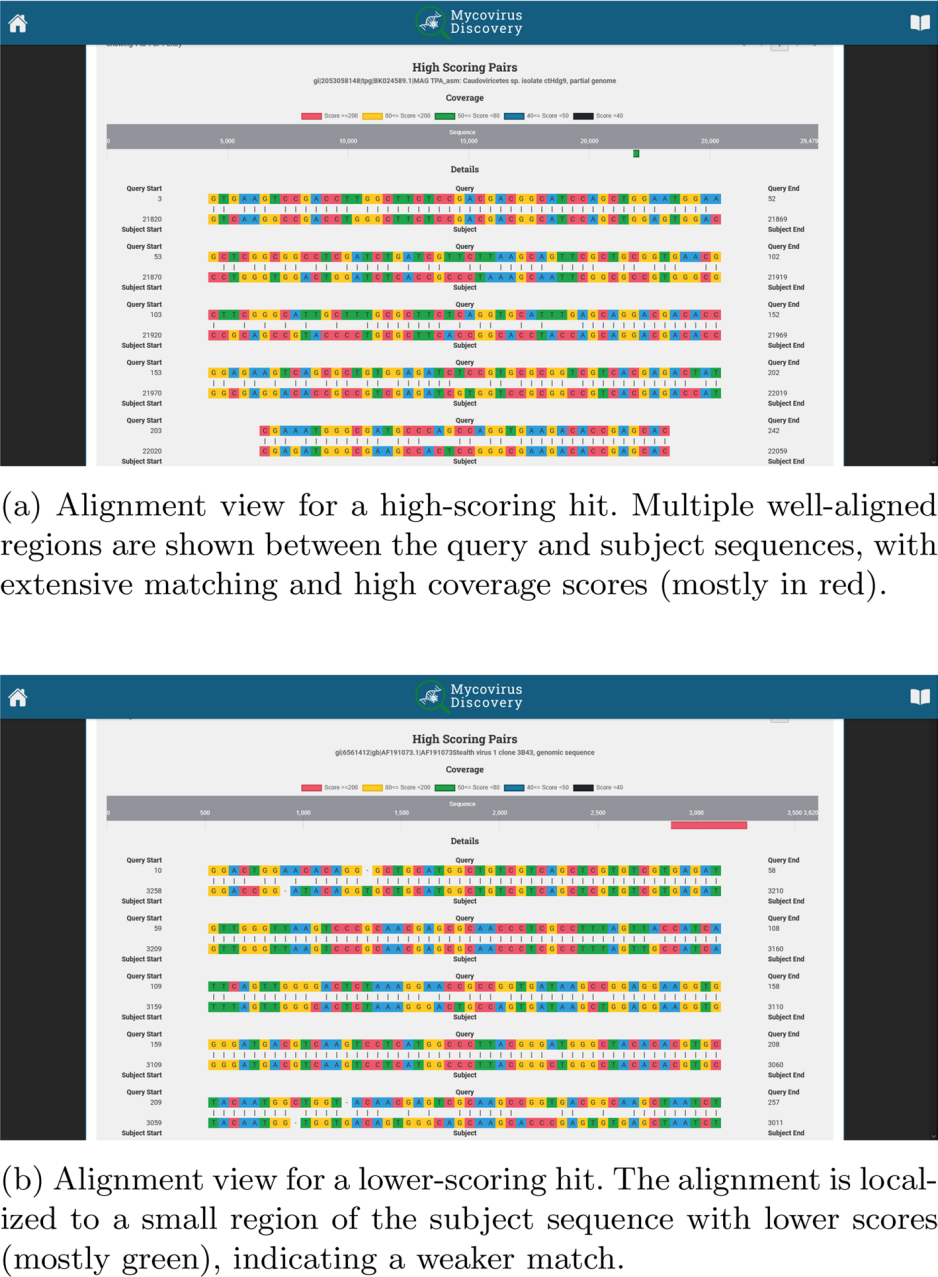
Detailed Report For each contig, the application visualizes the alignment between the query and the subject sequence. Color-coded regions indicate scoring ranges, with red to yellow indicating high score and green to blue representing lower score. Top panel: alignment view for a low scoring query. Bottom panel: alignment view for a high scoring query

In Fig. 7, the high-scoring pairs section presents detailed alignments for these pairs, using colour-coded sequences to show matches and mismatches between the query sequence and the subject sequence. This section provides a clear visual representation of the alignment, highlighting similarities and differences. The alignment of the low scoring query is localized to a small region of the subject sequence, indicating a weaker match in green (top panel). The alignment of the high scoring query to the subject sequence is more extensive Multiple well-aligned regions are shown between the query and subject sequences, indicating a stronger match in red. Finally, the researcher can easily download all results in XML format via the “DOWNLOAD ALL” button, for further exploration.

## Conclusion and further research

In this section, we summarise the unique contributions of our approach, its limitations, and areas for future exploration. DiscMycoVir successfully identified viral sequences present in the RNA-seq data derived from the transcriptome of a *C. auris* isolate. In theory, our pipeline can detect viruses from other organisms’ transcriptomes. Following a potential match of the contig used as query to a viral subject, the user should investigate further whether a replicating mycovirus is present in the *C. auris* isolate, and the match did not arise from contamination of the original sample or inaccurate information in the database used. Indications of a real mycovirus infection are multiple matches of contig queries covering the full genome of the viral subject. The user is also advised to perform an alignment of the processed reads to the viral subject. Our tool is limited in that can only detect mycoviruses that are not integrated in the fungal genome or present in DNA preparations used in genomic studies: RNA mycoviruses that undergo reverse transcription and DNA mycoviruses will not be detected with our pipeline. Another limitation stems from the RNA-seq data used and whether library preparation allows for inclusion of mycovirus sequences: for instance, processes that lead to discarding sequences without poly(A) tails may result in a paucity of mycoviruses in the library.

Further development of our pipeline could involve refining the approach to include the detection of reverse-transcribing RNA and DNA mycoviruses. By incorporating these additional viral types, the pipeline would not only become more comprehensive but also more versatile in identifying a wider array of mycoviruses. This enhancement would significantly broaden the scope of biological research, potentially leading to new insights into the detection and analysis of these complex viruses.

## Data Availability

The source code for the proposed system, along with installation and implementation instructions, is available at the following GitHub link: https://github.com/abompotas/DiscMycoVir. The platform can be accessed at http://discmycovir.imslab.gr:8000 (Accessed on 19/8/2024). Project name: Mycovirus Discovery (DiscMycoVir), Project home page: DiscMycoVir, Operating system(s): Linux, Programming language: Bash, Python, TypeScript, HTML, SCSS, Other requirements: Docker, License: MIT, Any restrictions to use by non-academics:–.

## References

[1] Myers JM, Bonds AE, Clemons RA, Thapa NA, Simmons DR, Carter-House D, et al. Survey of early-diverging lineages of fungi reveals abundant and diverse mycoviruses. mBio. 2020;11(5):e02027-20. 10.1128/mBio.02851-20. (**Erratum in: mBio. 2020;11(6):e02851-20**). 32900807 10.1128/mBio.02027-20 PMC7482067

[2] Kotta-Loizou I. Mycoviruses and their role in fungal pathogenesis. Curr Opin Microbiol. 2021;63:10–8. 10.1016/j.mib.2021.05.007. arXiv:3410.2567. 34102567 10.1016/j.mib.2021.05.007

[3] Harrison PW, Amode MR, Austine-Orimoloye O, Azov A, Barba M, Barnes I, et al. Ensembl 2024. Nucleic Acids Res. 2023;52(D1):D891–9. 10.1093/nar/gkad1049. 10.1093/nar/gkad1049 PMC10767893 37953337

[4] Altschul SF, Gish W, Miller W, Myers EW, Lipman DJ. Basic local alignment search tool. J Mol Biol. 1990;215(3):403–10. 2231712 10.1016/S0022-2836(05)80360-2

[5] Community TG. The Galaxy platform for accessible, reproducible and collaborative biomedical analyses: 2022 update. Nucleic Acids Res. 2022;50(W1):W345–51. 10.1093/nar/gkac247. 35446428 10.1093/nar/gkac247 PMC9252830

[6] López-Fernández H, Duque P, Vázquez N, Fdez-Riverola F, Reboiro-Jato M, Vieira CP, et al. SEDA: a desktop tool suite for FASTA files processing. IEEE/ACM Trans Comput Biol Bioinf. 2022;19(3):1850–60. 10.1109/TCBB.2020.3040383. 10.1109/TCBB.2020.3040383 33237866

[7] Reboiro-Jato M, Pérez-Rodríguez D, Silva M, Vila-Fernández D, Vieira C, Vieira J, et al. SEDA 2024 update: enhancing the SEquence DAtaset builder for seamless integration into automated data analysis pipelines. BMC Bioinform. 2024;05:25. 10.1186/s12859-024-05818-2. 10.1186/s12859-024-05818-2 PMC11131258 38802733

[8] Andrews S. FastQC: a quality control tool for high throughput sequence data. [Online]. Available online at: http://www.bioinformatics.babraham.ac.uk/projects/fastqc/. Available from: http://www.bioinformatics.babraham.ac.uk/projects/fastqc/.

[9] Bolger AM, Lohse M, Usadel B. Trimmomatic: a flexible trimmer for Illumina sequence data. Bioinformatics. 2014;30(15):2114–20. 10.1093/bioinformatics/btu170. arXiv:2469.5404. 24695404 10.1093/bioinformatics/btu170 PMC4103590

[10] Grabherr MG, Haas BJ, Yassour M, Levin JZ, Thompson DA, Amit I, et al. Full-length transcriptome assembly from RNA-Seq data without a reference genome. Nat Biotechnol. 2011;29(7):644–52. 10.1038/nbt.1883. 21572440 10.1038/nbt.1883 PMC3571712

[11] Li H, Durbin R. Fast and accurate short read alignment with Burrows–Wheeler transform. Bioinformatics. 2009;25(14):1754–60. 19451168 10.1093/bioinformatics/btp324 PMC2705234

[12] Li H. Aligning sequence reads, clone sequences and assembly contigs with BWA-MEM; 2013. arXiv preprint arXiv:1303.3997.

[13] Sayers E, Beck J, Bolton E, Brister J, Chan J, Comeau D, et al. Database resources of the National Center for Biotechnology Information. Nucleic Acids Res. 2023;52(D1):D33–43. 10.1093/nar/gkad1044. 10.1093/nar/gkad1044 PMC10767890 37994677

[14] Merkel D. Docker: lightweight linux containers for consistent development and deployment. Linux J. 2014;2014(239):2.

[15] Grinberg M. Flask web development: developing web applications with python. O’Reilly Media, Inc.; 2018.

[16] Ionic Framework.: Ionic Documentation. Accessed: 2024-07-02. Available from: https://ionicframework.com/docs.

[17] Jain N, Bhansali A, Mehta D. AngularJS: a modern MVC framework in JavaScript. J Glob Res Comput Sci. 2014;5(12):17–23.

[18] Axmark D, Widenius M.: MySQL 5.7 Reference Manual. Redwood Shores, CA: Oracle. Available online at http://dev.mysql.com/doc/refman/5.7/en/index.html. Available from: http://dev.mysql.com/doc/refman/5.7/en/index.html.

[19] Chybowska AD, Childers DS, Farrer RA. Nine things genomics can tell us about *Candida auris*. Front Genet. 2020;11:351. 10.3389/fgene.2020.00351. 32351544 10.3389/fgene.2020.00351 PMC7174702

